# Arthroscopic Bristow-Latarjet Combined With Bankart Repair Restores Shoulder Stability in Patients With Glenoid Bone Loss

**DOI:** 10.1007/s11999-014-3691-x

**Published:** 2014-06-19

**Authors:** Pascal Boileau, Charles-Édouard Thélu, Numa Mercier, Xavier Ohl, Robert Houghton-Clemmey, Michel Carles, Christophe Trojani

**Affiliations:** Department of Orthopaedic Surgery and Sports Traumatology, Hôpital de L’Archet 2, University of Nice Sophia-Antipolis, 151 route de St Antoine de Ginestière, 06202 Nice, France

## Abstract

**Background:**

Arthroscopic Bankart repair alone cannot restore shoulder stability in patients with glenoid bone loss involving more than 20% of the glenoid surface. Coracoid transposition to prevent recurrent shoulder dislocation according to Bristow-Latarjet is an efficient but controversial procedure.

**Questions/purposes:**

We determined whether an arthroscopic Bristow-Latarjet procedure with concomitant Bankart repair (1) restored shoulder stability in this selected subgroup of patients, (2) without decreasing mobility, and (3) allowed patients to return to sports at preinjury level. We also evaluated (4) bone block positioning, healing, and arthritis and (5) risk factors for nonunion and coracoid screw pullout.

**Methods:**

Between July 2007 and August 2010, 79 patients with recurrent anterior instability and bone loss of more than 20% of the glenoid underwent arthroscopic Bristow-Latarjet-Bankart repair; nine patients (11%) were either lost before 2-year followup or had incomplete data, leaving 70 patients available at a mean of 35 months. Postoperative radiographs and CT scans were evaluated for bone block positioning, healing, and arthritis. Any postoperative dislocation or any subjective complaint of occasional to frequent subluxation was considered a failure. Physical examination included ROM in both shoulders to enable comparison and instability signs (apprehension and relocation tests). Rowe and Walch-Duplay scores were obtained at each review. Patients were asked whether they were able to return to sports at the same level and practice forced overhead sports. Potential risk factors for nonhealing were assessed.

**Results:**

At latest followup, 69 of 70 (98%) patients had a stable shoulder, external rotation with arm at the side was 9° less than the nonoperated side, and 58 (83%) returned to sports at preinjury level. On latest radiographs, 64 (91%) had no osteoarthritis, and bone block positioning was accurate, with 63 (90%) being below the equator and 65 (93%) flush to the glenoid surface. The coracoid graft healed in 51 (73%), it failed to unite in 14 (20%), and graft osteolysis was seen in five (7%). Bone block nonunion/migration did not compromise shoulder stability but was associated with persistent apprehension and less return to sports. Use of screws that were too short or overangulated, smoking, and age higher than 35 years were risk factors for nonunion.

**Conclusions:**

The arthroscopic Bristow-Latarjet procedure combined with Bankart repair for anterior instability with severe glenoid bone loss restored shoulder stability, maintained ROM, allowed return to sports at preinjury level, and had a low likelihood of arthritis. Adequate healing of the transferred coracoid process to the glenoid neck is an important factor for avoiding persistent anterior apprehension.

**Level of Evidence:**

Level IV, therapeutic study. See Instructions for Authors for a complete description of levels of evidence.

## Introduction

Multiple recurrent shoulder subluxations or dislocations may be responsible for both erosion of the anterior glenoid rim and irreversible stretching of the anteroinferior capsule [7, 51]. The surgical treatment of recurrent anterior shoulder instability associated with severe glenoid defects and capsular deficiency remains challenging [8, 13, 32, 38, 39]. In these circumstances, the combined capsular and bone deficiency would render performing a Bankart procedure difficult and unreliable [7, 10, 20, 31, 42, 47, 49, 51]. It has been shown that patients with severe glenoid bone defects, involving more than 20% of the glenoid surface, risk failure after isolated arthroscopic soft tissue (ie, Bankart) procedures [7–9, 13, 15, 26, 32, 34, 38, 39, 51]. Glenoid reconstruction with auto- or allograft is another surgical option, but it only addresses part of the problem and does not treat the irreversible elongation of the inferior glenohumeral ligament [25, 39].

The Bristow-Latarjet procedure, transferring the coracoid and attached conjoined tendon to the anterior glenoid, is currently used to treat recurrent anterior instability in patients with severe glenoid bone loss and capsular deficiency [1, 10, 14, 16, 19, 20, 22, 23, 30, 31, 42, 47, 49]. In these procedures, the coracoid bone block is osteotomized, passed through the subscapularis muscle, and fixed at the site of the glenoid defect, either in the standing position with one screw (Bristow procedure) [18, 33, 49] or in the laying position with two screws (Latarjet procedure) [29, 37, 48]. Among the benefits of the Bristow-Latarjet procedure is that, by transferring the coracoid process and the conjoined tendon onto the anterior neck of the glenoid, this procedure not only restores the glenoid bone defect but also reinforces the weak and definitively stretched anteroinferior capsule [20, 23, 29, 37, 48].

Several recent investigations have reported good or excellent outcomes with the Bristow and Latarjet procedures [1, 3, 14, 19, 20, 22, 23, 30, 31, 42, 47, 49]. Both procedures provide a low rate of recurrent instability, a high rate of return to sports at preinjury level, and a high rate of patient satisfaction. However, the Bristow-Latarjet procedure remains controversial (albeit mainly in the North American literature) because of possible postoperative loss of external rotation, hardware failures, and development of osteoarthritis [13, 43, 54, 55]. Overhead throwing athletes may be particularly intolerant of external rotation loss and unable to return to high-performance competition [13].

**Fig. 1A–B:**
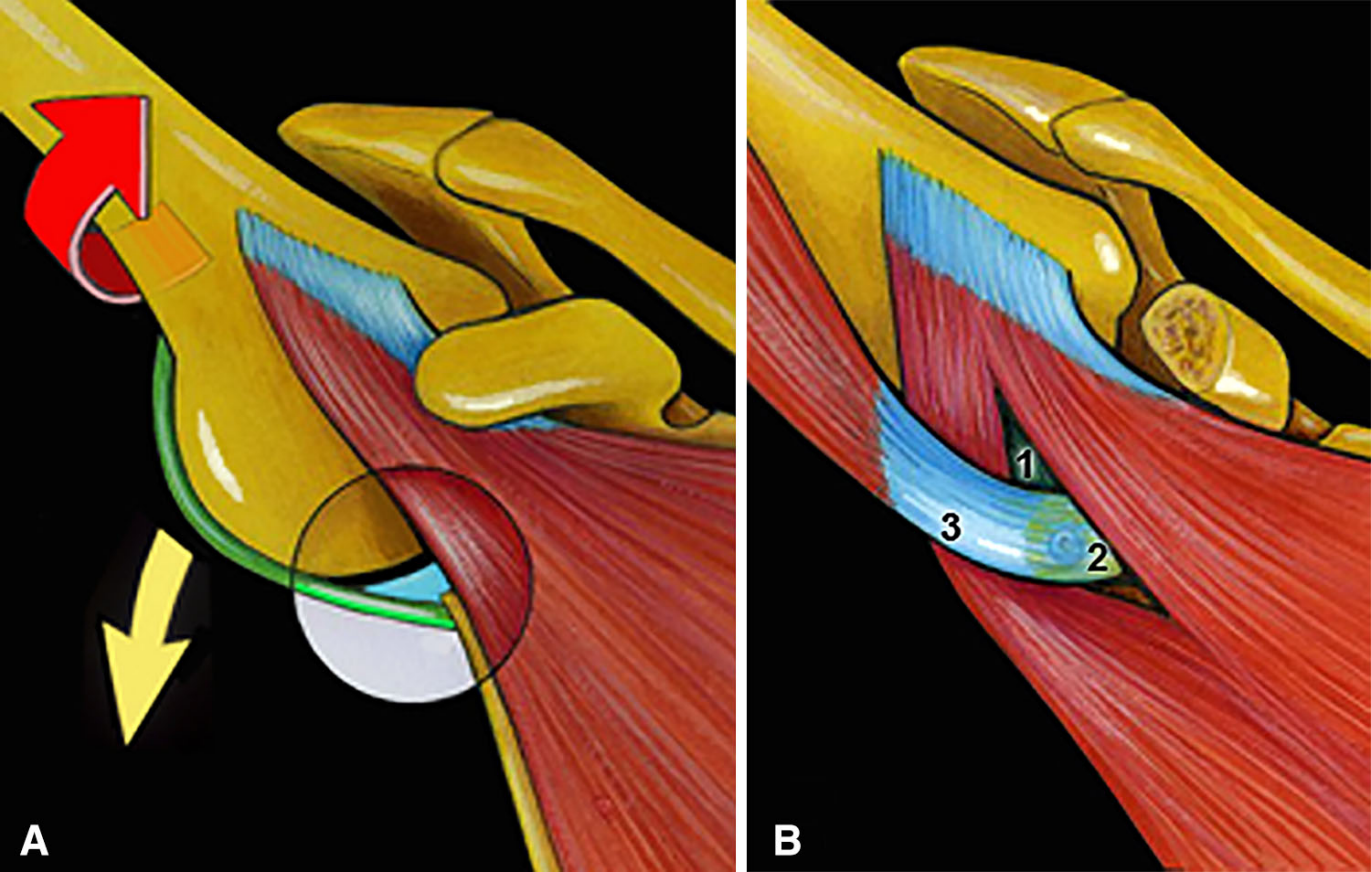
The benefits of the Bristow-Latarjet-Bankart procedure are illustrated. (**A**) In the throwing (abduction external rotation) position, the subscapularis slides over the equator, leaving only the weak detached anteroinferior labrum and stretched capsule to stabilize the humeral head. The 5 o’clock point is truly the vulnerable point of the glenoid rim. (**B**) By performing two procedures (Bankart + Bristow-Latarjet), a triple blocking of the shoulder is obtained (2B3 procedure): (1) bumper (or Bankart) effect, (2) bony (or bone block) effect, and (3) belt (or biceps) effect.

**Fig. 2A–C:**
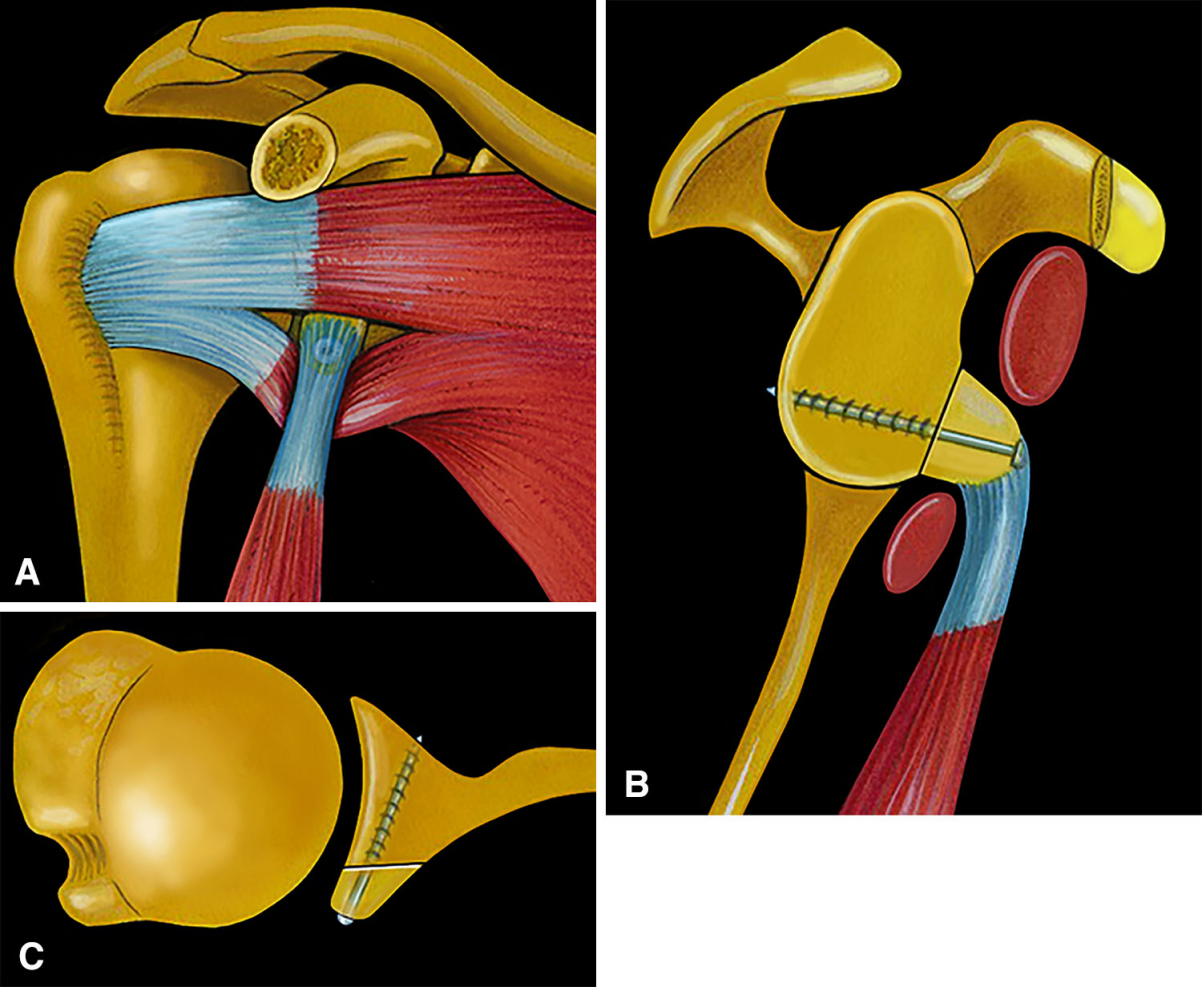
The surgical principles of the Bristow-Latarjet procedure are illustrated. (**A**) The osteotomized tip of the coracoid process is passed through the subscapularis split, turned 90° laterally, and fixed on the glenoid neck with a screw; the conjoined tendon provides both a belt (or sling) effect and dynamic tensioning of the lower part of subscapularis when the arm is abducted, whereas the bone graft restores (**B**) the width and (**C**) the concavity of the glenoid surface.

Since 2007, we have been using an arthroscopic modification of the Bristow-Latarjet procedure in association with a Bankart repair for patients with recurrent anterior shoulder instability associated with severe glenoid deficiency [4, 5]. This technique has been termed the 2B3 procedure because, by performing the two procedures (Bristow-Latarjet + Bankart), a triple locking of the shoulder is obtained (Fig. 1): (1) the transferred coracoid bone block restores osseous architecture of the glenoid rim (bony effect); (2) the transferred conjoined tendon creates a dynamic belt that reinforces the weak anteroinferior capsule by lowering the inferior part of the subscapularis when the arm is abducted and externally rotated (seat belt effect); and (3) the labral repair recreates the anterior bumper and protects the humeral head from direct contact with the coracoid bone graft (bumper effect) (Fig. 2). In a previous study, we reported the early encouraging results of this arthroscopic procedure [4, 5].

In the present study, we evaluated this procedure with a larger series of patients and longer (minimum 2 years) followup. Specifically, we determined whether this procedure would (1) restore shoulder stability in this selected subgroup of patients, (2) without decreasing mobility, and (3) allow patients to return to sports at preinjury level. We also evaluated bone block positioning, healing, and arthritis, (5) risk factors for nonunion and coracoid screw pullout, and (6) complications and reoperations.

## Patients and Methods

### Study Design

To evaluate this arthroscopic procedure, we performed a retrospective study performed with some data prospectively gathered.

The decision to perform an arthroscopic Bristow-Latarjet-Bankart procedure was based on two criteria: (1) an Instability Severity Index Score (ISIS) of greater than 3 points, which predicts a high risk of failure with an arthroscopic Bankart repair alone [2], and (2) the presence of a severe glenoid bone defect (> 20% of the glenoid surface as measured on preoperative CT scan according to Sugaya et al. [45] and confirmed at arthroscopy, according to Burkhart et al. [11]). The following patients were excluded from the investigation: (1) those with acute (first-time) anterior dislocation or subluxation; (2) those with voluntary or multidirectional instability; (3) those with isolated soft tissue lesions treated with an isolated capsulolabral repair [7]; (4) those with glenoid fractures (but no bone loss, ie, bony Bankart) treated with bone fragment reattachment and capsulolabral repair; (5) those with an isolated Hill-Sachs lesion (without glenoid bone loss) treated with Hill-Sachs remplissage and capsulolabral repair [6]; and (6) those with previously failed anterior stability repair.

Between July 2007 and August 2010, 79 patients met the above-defined criteria. Nine patients (11%) were either lost before 2 years of followup or had incomplete data, leaving a cohort of 70 patients available for review at a minimum of 2 years (mean, 35 months, range, 24–60 months).

### Study Population

The 70 patients (56 male, 14 female) underwent the all-arthroscopic combined procedure. The mean ± SD age at the time of operation was 24 ± 7.2 years (range, 17–45 years). The dominant arm was involved in 39 patients (56%). The mean duration of instability symptoms before surgery was 65 months (range, 7–480 months); 17 patients (24%) had recurrent dislocations, 20 (29%) had recurrent subluxations, and 33 (47%) had both subluxations and dislocations. The mean number of instability episodes was variable: five episodes (range, two to 100) for the patients with dislocations, 58 episodes (range, three to 100) for the patients with subluxations, and 42 episodes (range, three to 300) for the patients with dislocations and subluxations. Sixty-four patients (91%) were involved in sports, with 59 (84%) participating in high-risk sports involving collision and/or throwing (eg, rugby, handball, basketball, judo, etc); 31 patients (44%) were playing at a competitive level. The mean ISIS [2] in the patient population was 5.4 ± 1.77 (range, 3–10). All patients had glenoid bone loss of greater than 20% of the glenoid surface on preoperative CT scans: 50 patients (71%) had glenoid erosion with complete resorption of the bone fragment and 20 patients (29%) had a residual bone fragment. In addition, 61 patients (87%) had an impacted fracture of the humeral head (Hill-Sachs lesion). Patients were followed prospectively, and three subspecialty-trained shoulder surgeons (CET, NM, XO) independent of the operating surgeon performed the last clinical evaluation and review. The risks and benefits of the arthroscopic procedure were explained to the patients and they were aware that their data could be used for research purposes; all gave written, informed consent.

### Surgical Technique and Perioperative Management

**Fig. 3:**
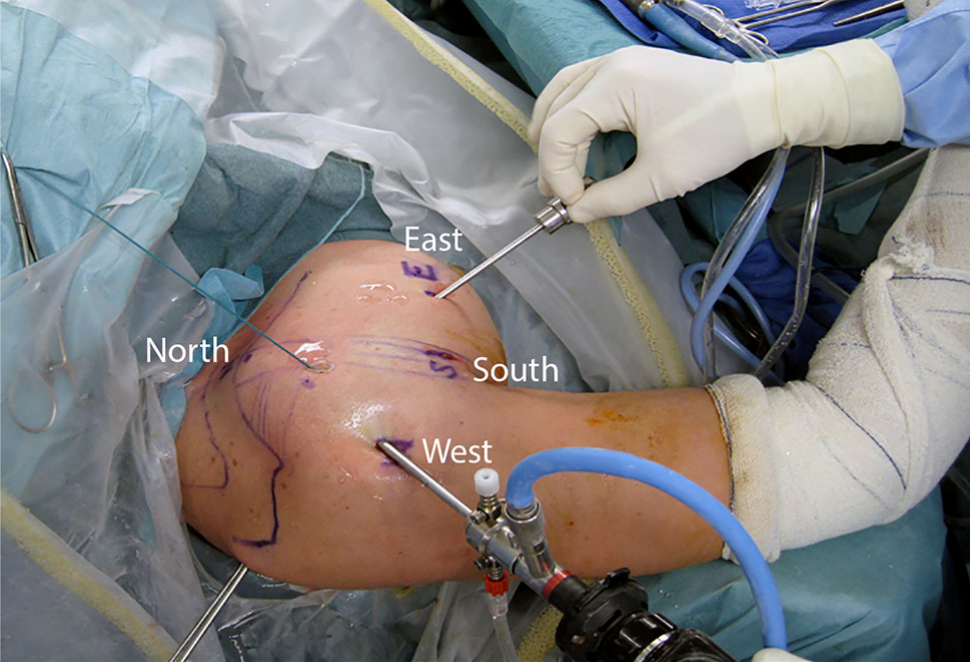
Patient and portal positioning for the Bristow-Latarjet-Bankart procedure is shown.

**Fig. 4A–C:**
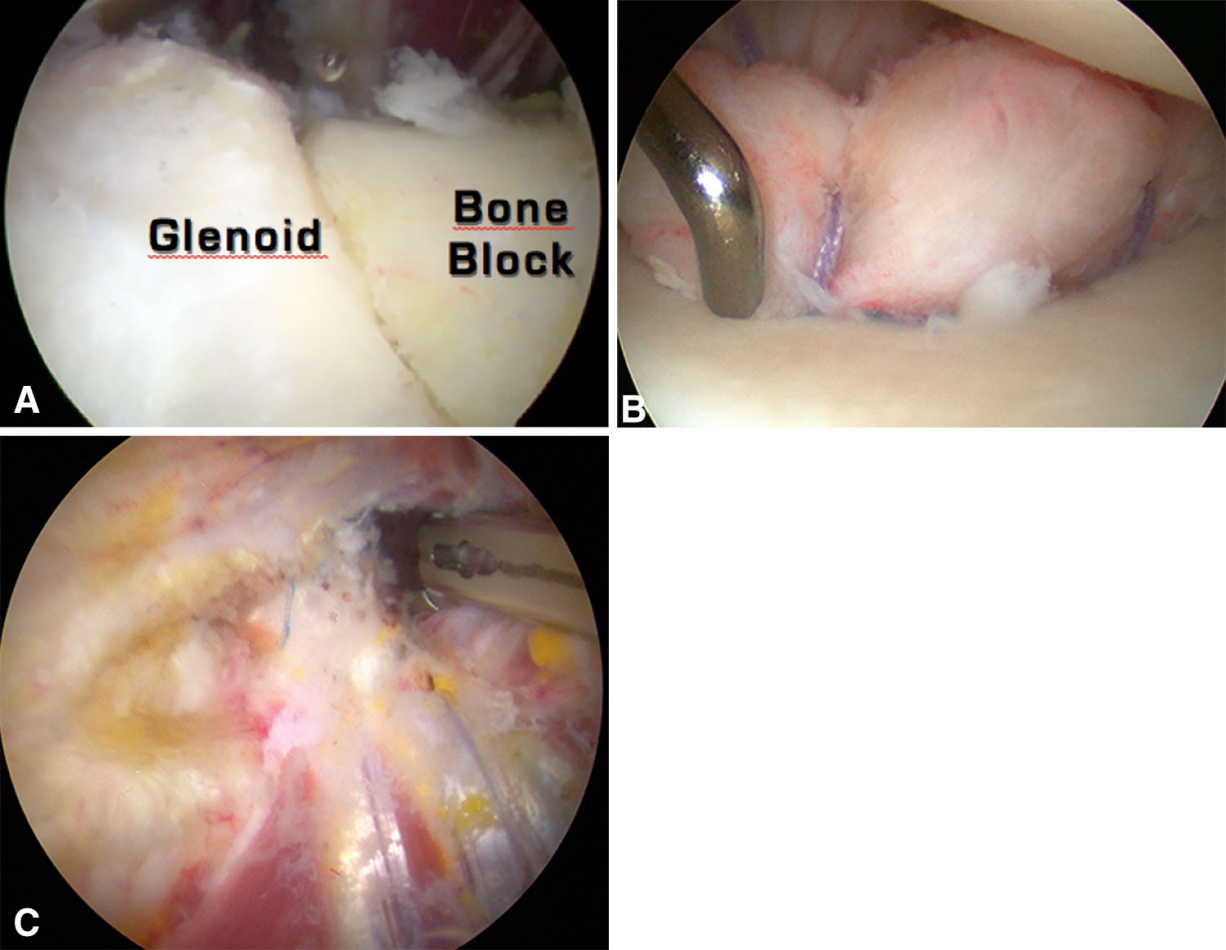
Arthroscopic views of the Bristow-Latarjet-Bankart procedure are shown. (**A**) An intraarticular view shows the coracoid graft positioned standing, fixed with a screw below the equator and flush to the glenoid surface (bony effect). (**B**) Associated capsulolobral repair (using two to three suture anchors) places the bone block in an extraarticular position and improves the concavity of the glenoid surface (bumper effect). (**C**) An extraarticular view shows the transferred conjoint tendon passing through the subscapularis muscle to reinforce the weak and stretched anteroinferior capsule (belt or sling effect).

The surgical technique has been previously described and will not be detailed here [4, 5]. All procedures were performed arthroscopically by the senior author (PB) with the patient in the beach chair position. In addition to the standard posterior and anterior portals, four additional anterior portals were used, on all sides of the coracoid process: north, south, west, and east [5] (Fig. 3). The technique was comprised of five operative steps, all performed arthroscopically. First, after detachment of the anterior labrum, the glenoid neck was abraded using a burr, and a screw hole was predrilled from posterior to anterior, using a specific glenoid guide (Smith & Nephew, Inc, Memphis, TN, USA) and a specific double K-wire (male and female); the female K-wire was left in place. Second, the coracoid process was predrilled with the help of another coracoid guide (Smith & Nephew, Inc) before insertion of a 4.0-mm cannulated screw, and the distal 1.5 cm of the coracoid was osteotomized using a motorized saw. Third, a cable loop was passed through the osteotomized coracoid and screw to retract it medially and inferiorly; this allowed identification of the axillary nerve and division of the subscapularis muscle belly in line with its fibers at the superior 2/3-inferior 1/3 junction (ie, at the level of the glenoid K-wire). Fourth, the coracoid was transferred with the conjoined tendon through the subscapularis muscle after pulling the cable loop through the female glenoid K-wire from anterior to posterior; the coracoid bone block was then fixed in the standing position with the cannulated screw on the abraded glenoid neck, after a lateral rotation of 90° to match to the natural concavity of the glenoid. Fifth, the remaining capsule and labrum were then reattached to the glenoid rim with two or three suture anchors (ie, Bankart repair), leaving the bone block in an extraarticular position (Fig. 4).

**Fig. 5A–B:**
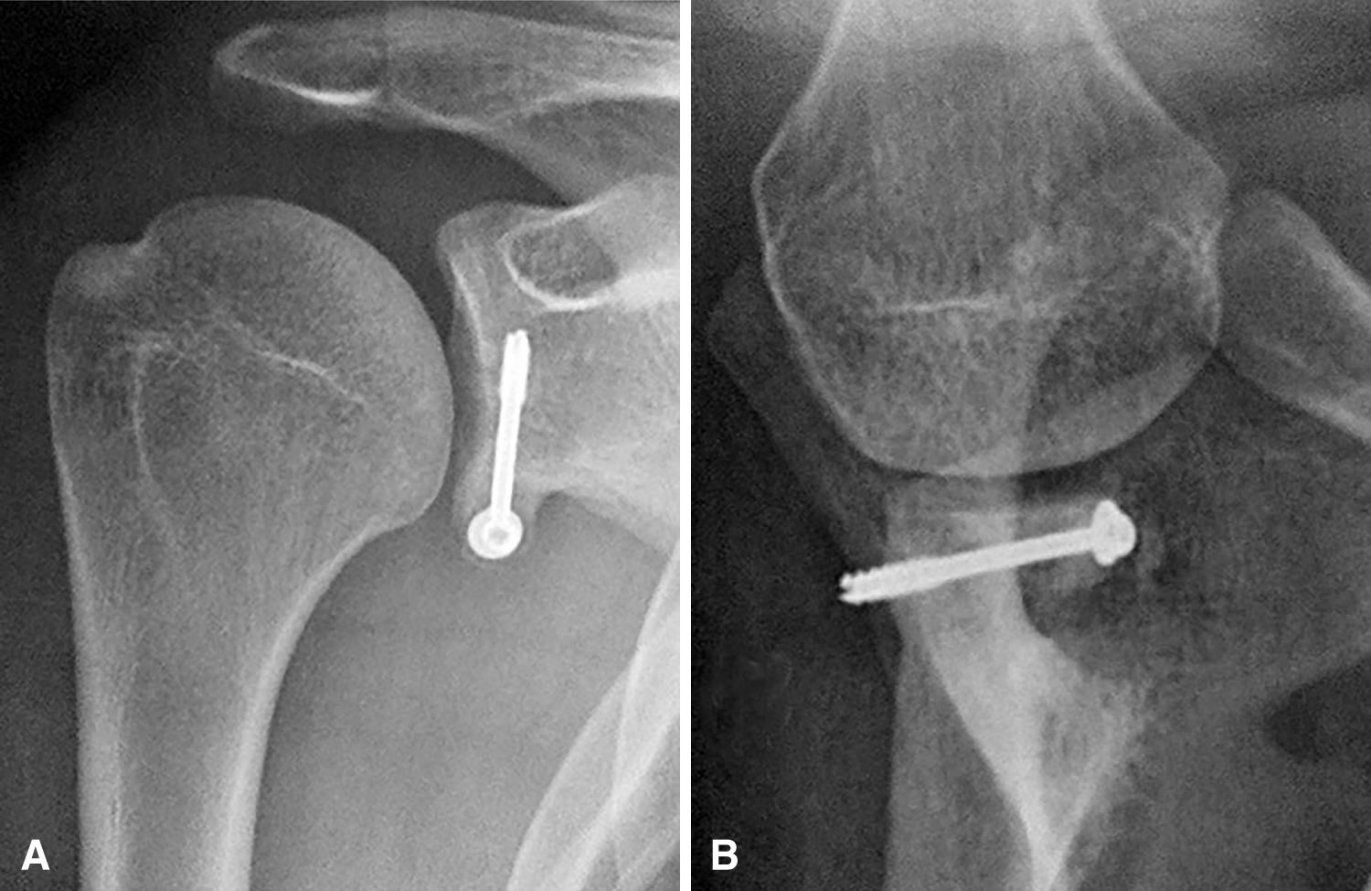
An example of coracoid graft optimal position and healing is shown. Postoperative (**A**) AP and (**B**) lateral radiographs demonstrate optimal positioning of the coracoid bone graft: below the equator and flush to the glenoid surface, restoring the concavity of the glenoid with perfect bone healing.

Postoperative radiographs were taken to confirm the correct positioning of the bone block at 5 o’clock and flush to the glenoid surface (Fig. 5). The patient was discharged from hospital the same day or the day after surgery. A neutral rotation sling was worn for the first 4 weeks. Self-directed rehabilitation with pendulum exercises began immediately (five times a day, 5 minutes each session). After 4 weeks, the sling was removed and formal rehabilitation with a physiotherapist was started. Hydrotherapy was recommended. No heavy lifting was allowed until 12 weeks postoperatively to ensure that solid bony union was obtained. Return to all types of sporting activities, including collision and contact-overhead sports, was allowed between 3 to 6 months postoperatively.

### Outcomes Assessment

All patients were examined clinically at 3, 6, and 12 months postoperatively and annually thereafter. Any postoperative dislocation or any subjective complaint of occasional to frequent subluxation was considered a failure. Physical examination included ROM in both shoulders and instability signs (apprehension test and relocation test). The Rowe [40] and Walch-Duplay [47] scores were obtained at each review. At latest followup, patients were questioned to determine whether they returned to sports at the preinjury level and whether overhead athletes returned to preinjury level of throwing.

**Fig. 6A–D:**
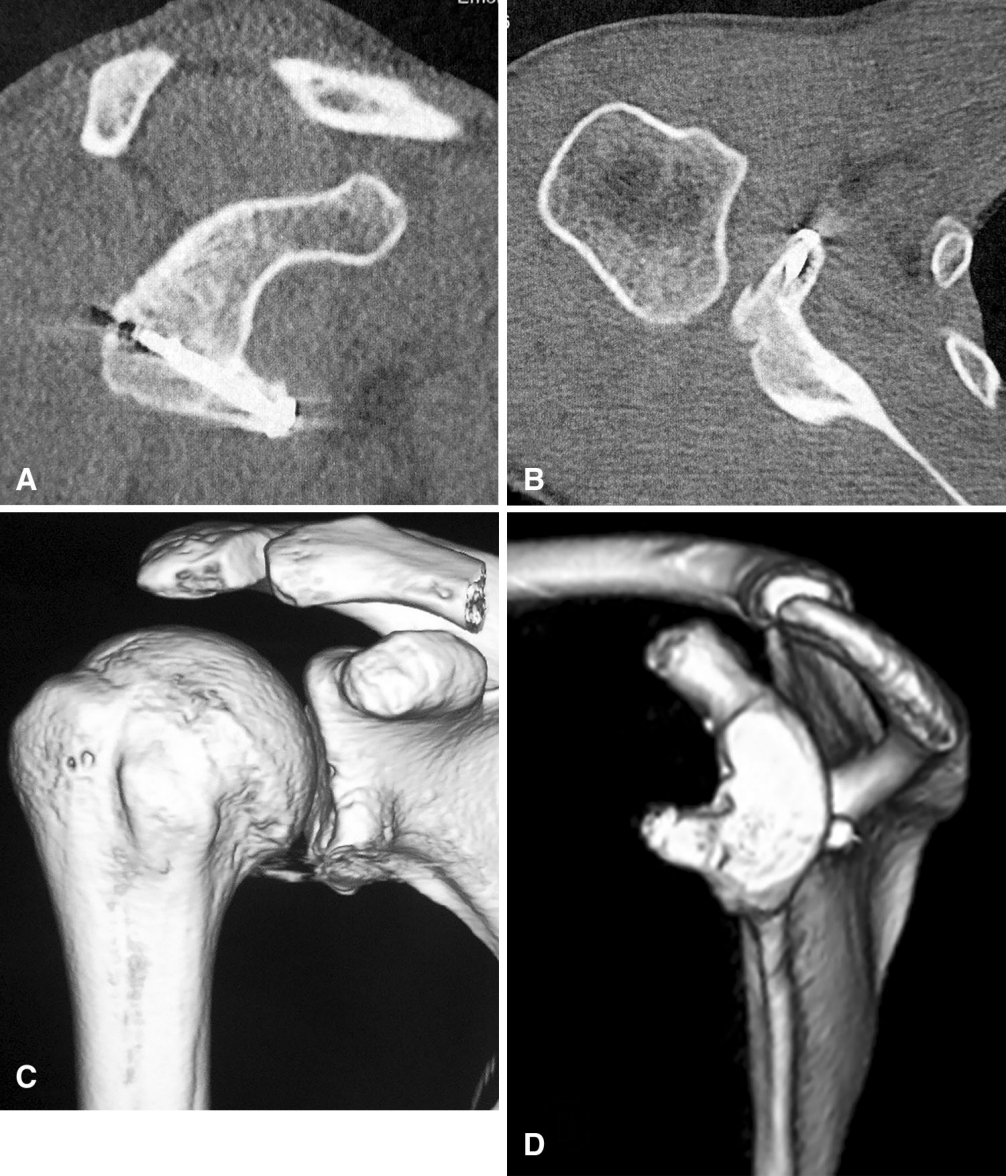
Postoperative (**A**) vertical and (**B**) axial CT scans and (**C**, **D**) three-dimensional CT reconstructions of the transferred coracoid bone graft demonstrate coracoid graft optimal positioning and healing.

Bone block positioning was evaluated using postoperative radiographs (AP and lateral views) and CT scans obtained 2 weeks after surgery. Graft healing was assessed at least 6 months postoperatively, using the same imaging studies (Fig. 6). The ideal position was defined as below the glenoid equator (in the vertical plane) and flush to the glenoid rim (in the horizontal plane) [1, 21, 47]. The bone block was judged to be too lateral if a step was visible beyond the level of the glenoid rim and too medial if it lay 5 mm or more medial to the rim. The bone block was considered to be malpositioned if any of the three observers judged it to be too medial or too lateral and/or too high. The length and direction of the screw in relation to the glenoid surface were analyzed using the CT scans. The subsequent radiographs and CT scans were also examined for bone block migration, fracture or lysis, hardware migration, or breakage. Glenohumeral degenerative osteoarthritis was evaluated using the Samilson and Prieto classification [41] on radiographs by the operating surgeon and an independent observer.

To determine potential factors related to bone block healing failure, we compared patients in whom the bone block had healed with patients in whom the bone block was either nonunited/migrated. We also assessed potential risk factors for nonhealing. In addition, we recorded any complications and reoperations.

### Statistical Analysis

To evaluate risks factors for nonhealing, we used Fisher variance test, with a multivariate analysis. The level of significance was set at p values of less than 0.05. Analysis was performed using StatView^®^ 5.0 (SAS Institute, Inc, Cary, NC, USA).

## Results

### Stability

The procedure consistently restored shoulder stability in this group of patients. At a mean followup of 35 months, 69 of 70 (98%) patients had a stable shoulder and only one (2%) experienced a redislocation. On clinical examination at latest followup, 13 patients (19%) had some remaining apprehension when testing the shoulder with the arm in the throwing position (positive anterior apprehension test). The mean Rowe score was 89.7 ± 14.4 (range, 65–100) and the mean Walch-Duplay score was 90 ± 12.5 (range, 50–100) (Table 1). Sixty-two patients (88%) had good or excellent results according to both scores.

**Table 1:** Functional results according to the Walch-Duplay and Rowe scoring systems

Scoring system	Score (points)
Walch-Duplay	
Sport or activity (maximum score, 25)	22.8 ± 5.6
Stability (maximum score, 25)	22.6 ± 4.7
Pain (maximum score, 25)	22.1 ± 4.5
Mobility (maximum score, 25)	23 ± 3.8
Total (maximum score, 100)	90.6 ±12.5
Rowe	
Function (maximum score, 50)	45.7 ± 7.9
Stability (maximum score, 30)	26.4 ± 6.7
Pain (maximum score, 10)	8.6 ± 2.5
Mobility (maximum score, 10)	8.9 ± 2
Total (maximum score, 100)	89.7 ± 14.4

Values are expressed as mean ± SD.

### Mobility

The mean active anterior elevation was 178° ± 4.9° (range, 160°–180°); mean external rotation with the arm at the side was 57° ± 18° (range, 20°–90°), compared to 66° ± 17° (range, 30°–90°) on the contralateral side. The mean loss of external rotation with the elbow at the body was 9° ± 8°. None of the patients were aware of any restriction in external rotation.

### Return to Sports

Fifty-eight (83%) returned to sports at the preinjury level. All the throwing athletes returned to their preinjury level of overhead throwing.

### Radiographic Findings

The bone graft positioning was accurate, with 63 of 70 (90%) being below the equator and 65 (93%) flush to the glenoid surface (Table 2).

**Table 2:** Coracoid bone graft position in relation to the glenoid on postoperative radiographs and early CT scans (performed 2 weeks after surgery)

Coracoid bone graft positioning	Number of shoulders (n = 70)
Vertical position	
Under the equator (correct graft position)	63 (90%)
At the equator (> 25% of bone block over equator line)	7 (10%)
Over the equator (> 50% of bone block over equator line)	0 (0%)
Horizontal position	
Flush to the glenoid surface (correct graft position)	65 (93%)
Too medial (> 5 mm medial to the glenoid rim)	2 (3%)
Too lateral (> 5 mm lateral to the glenoid rim)	3 (4%)

At a mean of 33 months (range, 24–54 months) postoperatively, the bone block was healed in 51 patients (73%), the coracoid graft failed to unite in 14 (20%), and graft osteolysis was seen in five (7%). Four patients had an early postoperative bone block fracture due to inadequate centering of the screw in the bone block (Table 3).

**Table 3:** Coracoid bone graft healing on postoperative radiographs and late CT scans (performed at least 6 months after surgery)

Coracoid bone block healing	Number of shoulders (n = 70)	Number of screw migrations
United	51 (73%)	3
Nonunited	14 (20%)	
Fibrous union	5 (7%)	3
Migration	5 (7%)	9
Fracture	4 (3%)	
Lysed	5 (7%)	

The mean inclination angle between the screw and the glenoid surface was 20.4° ± 8.9° (range, 5°–32°); there was no violation of the articular surface by the screw in any patient (eg, no lateral screw). The screw was bicortical in 58 (83%) and unicortical (too short) in 12 (17%); in four, the screw was considered slightly too long (> 5 mm). Nine patients had a screw pullout (six with graft nonunion/migration and three with graft fibrous union); four had reoperation for screw removal.

At a mean followup of 35 months, 64 patients (91%) had no glenohumeral osteoarthritis on the radiographs. According to the classification of Samilson and Prieto [41], five patients had minor osteoarthritis (Grade 1) and one patient had moderate osteoarthritis (Grade 2) (Table 4); none were symptomatic enough as of the time of this writing to request treatment.

**Table 4:** Glenohumeral osteoarthritis according to the classification of Samilson and Prieto [41]

Glenohumeral osteoarthritis	Number of shoulders (n = 70)
No osteoarthritis	64 (91%)
Osteoarthritis	6 (9%)
Grade 1 (humeral osteophyte < 3 mm)	5 (7%)
Grade 2 (humeral osteophyte between 3 and 7 mm)	1 (2%)
Grade 3 (joint line narrowing)	0 (0%)

### Risk Factors for Nonhealing

**Fig. 7A–E:**
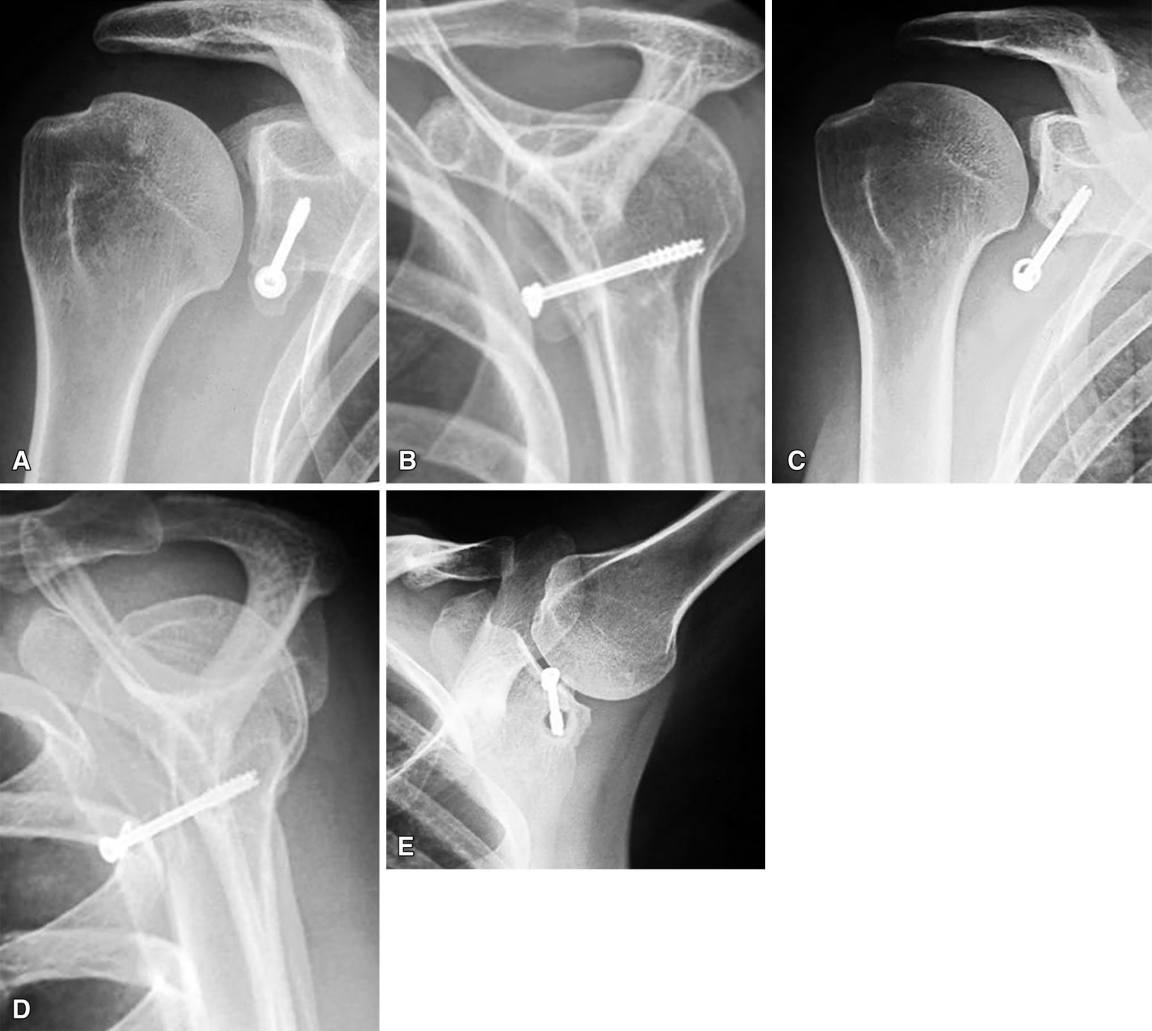
An example of coracoid graft nonunion is shown. Early postoperative (**A**) AP and (**B**) lateral radiographs demonstrate good positioning of the bone block but eccentric positioning of the screw in the bone block. (**C**) AP, (**D**) lateral, and (**E**) axillary radiographs at 3 years’ followup demonstrate loss of fixation and nonunion of the graft with screw pullout and a chamber of osteolysis. This patient was a 40-year-old woman and a heavy smoker.

When we compared the 51 patients whose bone block had healed with the 14 patients whose bone block was either nonunited or migrated (Fig. 7), we found that use of a unicortical (too short) screw (p < 0.01) and/or an overangulated screw (> 25°) (p < 0.01) relative to the glenoid surface was associated with poor bone block fixation and healing. Other factors significantly related to block nonunion/migration included patient age of more than 35 years (p < 0.03) and smoking (p < 0.01).

### Complications and Reoperations

Two patients had a temporary postoperative complication: the first had a postoperative hematoma along the axillary fold and arm that resorbed spontaneously after 6 weeks; the second patient had a transient musculocutaneous nerve palsy that recovered spontaneously by 6 months. None of these patients had any residual sequelae.

Four patients (6%) with bone block nonunion/migration were reoperated on, between 1 and 2 years after surgery because of pain related to screw pullout from the glenoid. The screw was removed under arthroscopy from intraarticular after detachment of the anterior labrum. No attempt was made to refix the coracoid fragment, and the labrum was reattached with sutures and anchors. In the fourth patient, the screw pullout was associated with a recurrent dislocation subsequent to secondary trauma (fall snowboarding). An arthroscopic Hill-Sachs remplissage was performed in addition to the Bankart repair and screw removal, leading to a stable shoulder at latest followup.

## Discussion

It has been suggested that arthroscopic Bankart repair alone cannot restore shoulder stability in patients with glenoid bone loss involving more than 20% of the glenoid surface [35], and we believe this is correct. Coracoid transposition to prevent recurrent shoulder dislocation according to Bristow-Latarjet may be an efficient surgical option in this context, although it remains controversial. In the present study, we found that an arthroscopic Bristow-Latarjet procedure with a concomitant Bankart repair restored shoulder stability in this selected subgroup of patients, without decreasing mobility, and allowed return to sports at preinjury level.

There are several limitations to this study. A randomized, controlled study was not used because we were developing a new arthroscopic technique. The evaluators were all members of the department and so were not blinded to the procedure employed. These two limitations might tend to increase the apparent efficacy of the procedure. On the other hand, our study has several strengths, including patient examination undertaken by observers not involved in the surgery; relatively good tracking of a young and active population, with only nine patients (11%) lost to followup before 2 years; and detailed radiographic and CT scan analysis performed postoperatively.

At a mean followup of 33 months, only one patient had a recurrence of shoulder instability in our series. Although the recurrence rate may increase with time, these results are as good as or even better than those previously reported for open Bristow-Latarjet coracoid transfer [1, 10, 14, 19, 20, 22–24, 30, 31, 42, 47, 49].

We believe the associated arthroscopic Bankart repair performed in our technique to be of paramount importance, contributing to shoulder stability (avoiding persistent subluxations) [14, 49, 52] and retaining capsular proprioceptive fibers, which are so important for sports [46, 52, 53]. In contrast to our technique, Lafosse et al. [27, 28] resected the anterior labrum and capsule in their arthroscopic Latarjet procedure. Although these authors claimed that the anterior capsule regenerated after its resection [27, 28], it is well known that the fibrous scar tissue loses all proprioceptive capacity. A recent study by Hovelius et al. [21] reinforced this concept, showing better results for stability when the Bristow-Latarjet procedure was associated with a capsular repair using suture anchors.

**Fig. 8A–B:**
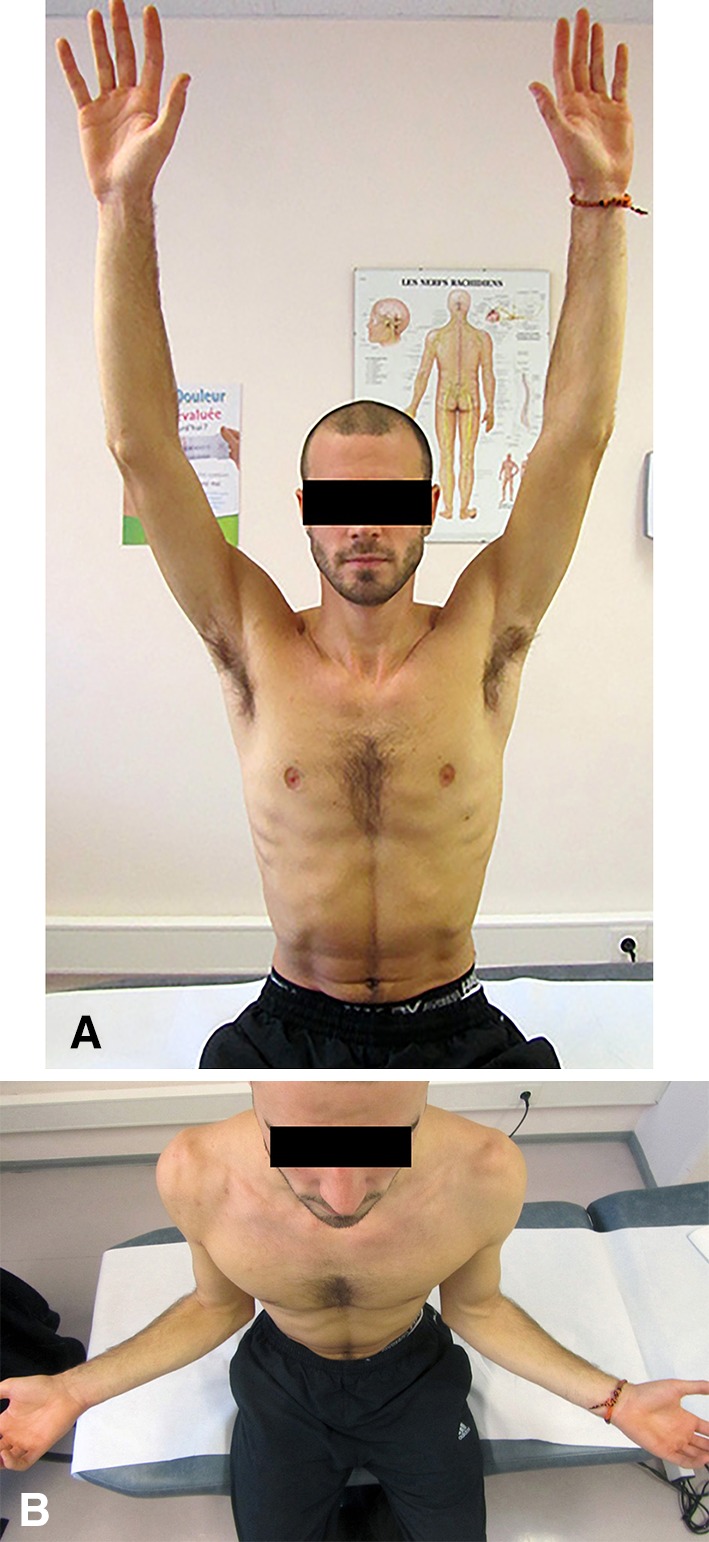
Photographs demonstrate restoration of shoulder mobility: (**A**) active elevation and (**B**) external rotation.

Postoperative restriction of external rotation has long been a major criticism of the Bristow-Latarjet procedure, leading some authors to contraindicate the procedure in overhead throwing athletes [44, 49, 54, 55]. However, our results dispute this criticism. The mean 9° loss of external rotation was not noticed by any of our patients, and all of the throwing athletes were able to resume sports (Fig. 8). We believe that this ROM results from some important intraoperative and postoperative details, including (1) splitting the subscapularis muscle parallel (2/3 up and 1/3 down) to its fibers rather than dividing it, (2) accurate placement of the coracoid graft flush to the articular surface, (3) fixing the bone block in the standing position (providing sufficient room for the lower 1/3 of the subscapularis muscle, thus decreasing the tethering effect, which may restrict external rotation), and (4) use of a neutral rotation brace postoperatively (which permits healing of the conjoined tendon in the muscular part of the subscapularis and not the tendinous part). The restoration of stability and mobility probably contributed to the high proportion of our patients who were able to return to sports at the preinjury level (83%).

Although similar to those reported by Hovelius et al. [20] with the open technique, our rates of bone block healing are disappointing. The coracoid graft failed to unite in 20% of patients and 13% had screw migration. Potential reasons for this might include the facts that (1) we used only one cannulated screw for the graft fixation (instead of two according to the Latarjet technique) and (2) we used a cannulated screw, which has inferior biomechanical performances compared to the recommended malleolar screw [20, 46]. As mentioned recently by Shah et al. [43], the thread depth of cannulated screws is less than that of noncannulated screws, which can affect purchase in the native scapula and may result in less compression of the graft.

Our data confirm that, although the Bristow-Latarjet is traditionally thought of as a bone block procedure, in reality part of the stability gained from this procedure is more likely to be attributable to the conjoined tendon sling (ie, dynamic sling or seat belt effect) [14, 17, 29, 37, 48–50]. Of the 14 patients with graft nonunion/migration in our series, only one had a recurrent traumatic dislocation. The discrepancy between shoulder stability and bone block healing has already been noted in some open technique series [1, 14, 19, 24, 30, 31, 47]. However, although the graft nonunion/migration does not compromise shoulder stability (thanks to the Bankart and seat belt effect of the transferred conjoined tendon, 2B2), failure to obtain graft healing is associated with a higher rate of persistent apprehension and a decreased return to sport participation. Our results confirm the previously reported finding that adequate healing of the transferred coracoid process to the glenoid neck is an important factor for avoiding persistent anterior apprehension [14, 20, 22, 49].

In identifying factors that could interfere with bone graft healing, we found that smokers and patients older than 35 years were at risk of failure of bony union; smokers are therefore poor candidates for such procedures. The detrimental effect of smoking on bone healing has been extensively reported in the literature and is nowadays well known [36]. It is now our current practice to convince patients to stop smoking before undergoing a coracoid transfer procedure. In common with other investigators, we found that technical errors such as the use of too short a screw (unicortical) or an overangulated screw (> 25°) was associated with poor bone block fixation and healing [12, 36, 47]. Strict patient selection (avoiding smokers) and the development and use of specific instruments and implants to firmly anchor the sectioned coracoid process to the anterior rim of the glenoid should decrease rates of bone block nonunion/migration in the near future.

Development of subsequent osteoarthritis after the Bristow-Latarjet procedure has been another major criticism, leading to some condemnation of the procedure in North America during the 1980s and 1990s [49, 54, 55]. In our series, 91% of the patients had no glenohumeral osteoarthritis at the most recent followup. Although longer followup is needed, we believe that both the labral repair and optimal graft placement (at 5 o’clock, flush with the articular surface) obtained arthroscopically helped to avoid development of glenohumeral arthritis. Arthroscopic visualization of the glenohumeral joint permits the Bankart repair and facilitates optimal positioning of the coracoid bone block in an extraarticular position. This prevents undesirable contact between the humeral head and the bone block-screw construct, avoiding pain and the development of osteoarthritis.

In conclusion, our results provide evidence that the combined arthroscopic Bristow-Latarjet and Bankart repair (2B3 procedure) effectively restores shoulder stability in patients with a severe glenoid and capsular deficiency. The arthroscopic procedure restores glenohumeral stability while preserving external rotation and resulting in a low likelihood of arthritis in the short term. As external rotation is so well preserved with this procedure, it even allows overhead throwing athletes to resume sports at their previous level. This arthroscopic technique provides good visualization of the glenohumeral joint, facilitating accurate placement of the transferred coracoid bone block, and allows secure labral repair. Graft nonunion/migration and screw pullout were observed in smokers, patients older than 35 years, and in association with technical errors. Although graft nonunion/migration does not compromise shoulder stability, it is associated with persistent anterior apprehension. Use of improved fixation techniques should provide more reliable healing of the transferred coracoid process to the glenoid neck. Surgeons should be aware that the arthroscopic Bristow-Latarjet-Bankart is a technically demanding procedure, with a steep learning curve, as much of the operation is performed outside of the glenohumeral joint in the anterior subdeltoid space. Thus, the technique can be recommended only to experienced orthopaedic surgeons with advanced arthroscopic skills and familiarity with the normal and abnormal anatomy encountered during the open Bristow-Latarjet procedure.
